# Nonconventional Hydrocolloids’ Technological and Functional Potential for Food Applications

**DOI:** 10.3390/foods11030401

**Published:** 2022-01-30

**Authors:** Sandra Viviana Medina-López, Carlos Mario Zuluaga-Domínguez, Juan Pablo Fernández-Trujillo, María Soledad Hernández-Gómez

**Affiliations:** 1 Instituto de Ciencia y Tecnología de Alimentos (ICTA), Universidad Nacional de Colombia, Bogota 111321, Colombia; svmedinal@unal.edu.co (S.V.M.-L.); mshernandez@unal.edu.co (M.S.H.-G.); 2 Facultad de Ciencias Agrarias, Universidad Nacional de Colombia, Bogota 111321, Colombia; cmzuluagad@unal.edu.co; 3 Department of Agronomical Engineering, Technical University of Cartagena, E-30203 Cartagena, Spain; 4 Instituto Amazónico de Investigaciones Científicas (SINCHI), Bogota 110311, Colombia

**Keywords:** polysaccharides, food carbohydrates, agrobiodiversity, starch, phycocolloids, alternative ingredients

## Abstract

This review aims to study the alternatives to conventional industrial starches, describing uncommon sources along with their technological characteristics, processing, and performance on food products. Minor components remaining after extraction play an important role in starch performance despite their low percentage, as happens with tuber starches, where minerals may affect gelatinization. This feature can be leveraged in favor of the different needs of the food industry, with diversified applications in the market being considered in the manufacture of both plant and animal-based products with different sensory attributes. Hydrocolloids, different from starch, may also modify the technological outcome of the amylaceous fraction; therefore, combinations should be considered, as advantages and disadvantages linked to biological origin, consumer perception, or technological performance may arise. Among water-based system modifiers, starches and nonstarch hydrocolloids are particularly interesting, as their use reaches millions of sales in a multiplicity of specialties, including nonfood businesses, and could promote a diversified scheme that may address current monocrop production drawbacks for the future sustainability of the food system.

## 1. Introduction

Colloidal systems are those with a complex multiphase structure, where particles of different kinds, collectively referred to as the disperse phase, scatter onto a homogeneous, continuous phase [1]. Water is a ubiquitous vital fluid, having a major share in the composition of almost every source of food found in nature; hence, the colloids whose continuous phase is made up by this liquid are known as hydrocolloids (HC) and are a frequent topic of research. HCs can arise from animal tissue, plant tissue, or microbial fermentation, and the available forms of the ingredients are diversified through physical and chemical alterations after the extraction [2].

Starches are highly appealing, common, advantageous, and versatile HCs, being a target of numerous methods of engineered modifications, and are used in a vast array of food products. The main sources of starch in the world today, also known as traditional or conventional, are corn, potatoes, rice, wheat, and cassava; these are considered monocrops that impose agricultural pressure in many ecosystems where they are not endemic or are insufficient to supply industrial demand, and, consequently, many other detriments are seen. The obligation to import the ingredient or modify edible landscapes to produce them without proper technologies to handle the supply chain may lead to a deterioration of native crop systems, thereby risking local food security. This risk is already projected to rise due to the current climate and health crisis globally, but especially in developing countries already facing many challenges [3,4]. An alternative way to promote sustainable agrobiodiverse systems is to promote local starch sources, which can differ structurally and functionally from traditional starches, opening up new possibilities for starch research and business.

Nonconventional HCs are extracted from diverse sources such as exudates or extracts from other seeds, biomass, mucilages, fruits, peels, barks, or even leaves from species that have not been extensively studied or grown, in contrast to those mentioned above. Examples of such compounds may be as diverse as the biology of the geographical research zone; not only plants but also algae or industrial byproducts can be harnessed in different regions of the world for food applications where the HCs are useful. The advantages of lesser-known HCs and starches must be highlighted for industrial purposes, considering the diverse strategic effects of its combination, as different configurations of food matrices may result in greater sensory appeal and improve the operational or economic benefits. Therefore, these have become an engaging topic of study. Findings on the versatility of HCs are closely related to their biological source, the assortment of their features, and the modification of their composition through further treatments. Other nonstarch HCs may compete with regular commercial staples, without detrimental effects on environmental and socioeconomic systems usually linked to monocrop systems [5,6,7]. Recognized experts in the field have brought up how the relevance of alternative HC goes far beyond the basic attributes regularly considered, impacting sustainability, the consumer experience, and cultural and social affairs [8]. This study reviews nonconventional sources of HCs, including a special category of starch sources as such popular rheology modifiers, with the corresponding characteristics making them suitable for food industry applications with health and marketing implications. When information is not available on these sources of HCs, a few updates containing literature examples of conventional HCs are provided in order to illustrate potential research directions using nonconventional ones.

## 2. HCs in Food Products

Overall, food HCs share a macromolecular character as polymers and the ability to shape intertwined networks that translate into macroscopic gels, modifying viscosity or thickening the aqueous medium they are added to [9]. At a molecular level, the polysaccharides or proteins typically making the disperse phase of these colloids have sizes between 1 to 1000 nm, and, as a natural consequence of the abundance of hydroxyl groups, interact through hydrogen bonds with surrounding water molecules to increase their affinity and turn them into hydrophilic compounds with the ability to overlap or entangle into diverse networks, modifying the rheology of the food complexes [10]. This property has been widely seized on for developments in the food industry, making HCs into alluring ingredients for both their viscosifying, stabilizing, or gelling action in the continuous quest to meet the consumers’ palatability requirements; and their role in bioactive protective compound, including edible coatings made possible on account of the unique technological features that this kind of additive provided [11]. Examples of colloidal foods can be found throughout the industry: droplets of water, oil, gas cells, and fat crystals are distributed evenly in many products such as bread, yogurts, sauces, meringues, and spreads to accomplish an overall structure that will be translated into a pleasant mouthfeel [12,13]. Depending on the state and proportion of the phases, colloids may be classified into solids or foams when the minor phase is composed of gas and the major phase is either in a solid or a liquid state, respectively; emulsions if both phases are liquid; solid emulsions or gels if solids are larger than liquids; and sols if liquids are greater than solids [1,14].

In general, HC substances such as gums, resins, mucilages, or starch, as plant-based ingredients, share the advantage of higher demand in comparison to their animal counterparts, on the account of their “safe green nature” and positive consumer feedback; although animal-sourced HCs such as chitosan, gelatin, and caseinates are an important field of research as substantial byproducts of the food industry, this review focuses on algae, plant-based, and some microbial sources, which are trending in markets and academia [15]. The most common or conventional hydrocolloids of great commercial relevance, defined by their origin, include [16,17,18]:

- Plant-based: tree cellulose; exudates such as gum Arabic, karaya, ghatti, and tragacanth; seeds such as guar, locust bean, tara, tamarind gum, or konjac mannan; tuber starches; and fruit pectin.
- Algal: brown seaweed alginate, red seaweed agar, and carrageenan.
- Microbial: curdlan, dextran, cellulose, xanthan, or gellan gum.
- Animal sourced: gelatin, caseinate, whey protein, and chitosan.

HCs are used as ingredients with a multiplicity of food uses, ranging from stabilizing emulsified drinks, desserts, or foams [19,20,21] to preventing oil absorption in chip making [22], fruit gelling [23,24,25], meat structuring [17,26,27], and food coating [11]. Ice cream, one of the most common complex colloidal food systems, bears fat globules, air bubbles, and ice crystals in an aqueous matrix that relies on HCs such as agar, plant exudates, and extracts, or even the recently studied basil seed gum, to stabilize the dispersed molecules, inhibiting crystal growth to achieve an enticing mouthfeel [10]. Restructured snacks are examples of products where HCs are of critical relevance as well, acting to create semisolids and gels from processed fruit pulps or meat derivates, whose combination with starches and gums mainly serves to attain the precise hardness, adhesiveness, cohesiveness, or chewiness necessary for consumer acceptance of fruit bars, jellies, gummies, sausages, and cold meats [23,26]. Fluid products where thickening or emulsification is also required, such as dressings, dairy, or plant-based drinks, take advantage of citrus-based pectin, animal gelatin, or algae alginates to modify the rheology of the liquids, having the capacity to bind water, flavor, and oil in products where suspending or, stabilization is also required to prevent beverage syneresis and improve consumer quality perception [28]. In desserts such as mousses or creams, HCs have become indispensable as “airy gels” of very soft texture are achieved through the synergy of hydrocolloid blends containing k-carrageenan, locust bean gum, agar, xanthan gum, and pectin, for example, and their interaction with sugar and dairy complexes [29]. Encapsulation of bioactive compounds is also an important task accomplished through the use of hydrocolloids, as in recent research using alternative *Prosopis alba* gums as polyelectrolytes to improve calcium alginate–chitosan beads for the delivery of fish oil [30].

Current advances have opened up ways of developing new convenience products that comply with the constantly evolving food industry, driven by both regulatory affairs and shifting consumer preferences, now increasingly focused on healthier foods to promote wellness [31]. Most HCs are extracted through a couple of simple operations applied to raw materials; this has the advantage of saving money to put toward further technological inputs, and also reassures consumers because of the “natural” label in contrast to “modified” HCs—being ingredients with limited processing and recognizable names [32].Novel formulations have goals of safety and meeting technological and sensory needs in food design, where additives that promote wellbeing prevail, while avoiding ingredients associated with detrimental effects for humans. Many HCs have also the advantage of being labeled as functional ingredients, as most of them are dietary fiber sources with several health claims linked to blood cholesterol regulation, the achievement of normal body weight, and reducing the postprandial glycemic response when consumed in the right amounts [33].

In some cases, distasteful end products result from the amount of additive required to achieve the technological function, and HCs may impart flavor or taste characteristics that might be noticeable in concentrations higher than 5% [17], hence making their use less appealing. However, most HCs perform their tasks in concentrations below 1%; in very few cases, such as gum arabic, they may make up 10% of the working mix [34]. When properly used, then, HCs might convey health advantages while enhancing palatability in several ways such as water binding/retaining, mimicking an oil impression in the mouth, correcting acidity, masking off flavors, and even reducing undesirable syneresis, as does the interaction of starch and cellulose derivatives, for example [35].

### 2.1. Starches

In spite of being a structural carbohydrate with textural, nutritional, and functional roles in food, starch is commonly regarded independently from other HCs because of its function as a reserve substance in plant tissues [27]. Millions of years ago, endosymbiosis gave rise to the first plastids, which led to cell survival reservoirs in the form of carbohydrates and, of particular concern to this review, we can trace the origins of starch production among algae and green plants [36]. The latter has turned into the main source of starch extraction, becoming a main raw material for the benefit of early *Homo sapiens* more than 300,000 years ago, and still today contributing greatly to human diets [37].

Some of the outstanding features of starches are their ready availability, abundance, and inexpensive nature; they result from the carbon fixation systems of autotrophic organisms, are molecularly built by amylose and amylopectin glucose polymers, and settle in granules of 1–100 μm in diameter [29,30]. Amylose constitutes less than one-third of the structure and reveals a linear assembly, with glucose residues binding through 1,4-α-glycosidic linkages on a 103–104 degree of polymerization, in contrast to branched amylopectin, which makes up nearly the remaining 70% of the granule and combines 1,4 and 1,6-α-glycosidic bonds with approximately 104–107 glucose units per molecule [4]. Exceptions to these patterns are seen in mutant genotypes dubbed “waxy” because of the appearance of the endosperm, whose granules consist of nearly 99% amylopectin; these are the opposite of 50–70% amylose “amylotype starches,” which result in opaque, stronger gels, in contrast to weak translucent waxy starch networks [32].

A structural analysis has revealed how amorphous or crystalline regions of the starch granules are shaped by the amount of bound water and amylose–amylopectin compactness, and has also pinpointed distinct structures thought to be exclusive to some plant groups. X-ray diffraction (XRD) patterns for granules of cereal starch are typically designated A types, while tuber-sourced starches are B type, and legumes have a mixture of both A and B crystals and are known as C type. However, investigations of nonconventional botanical starches have demonstrated how, although differentiated A- and B-type crystals may be located in the inner and outer part of a starch granule, respectively, allomorph distributions of C-type XRD patterns may also show A/B-type crystals in different granules of potato beans (*Apios fortunei*) and colored sweet potato starches [38], giving new insight into the diversity of molecular arrangements that starch granules may display where A-, B-, and C-granules coexist.

**Figure 1:**
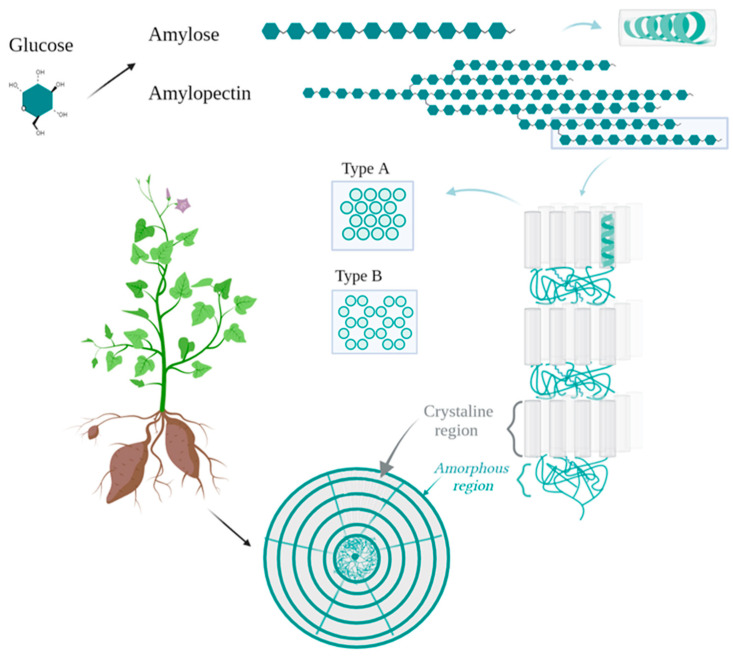
Starch granule structure, based on descriptions and graphics from [39,41]. Glucose linear and branched polymers are shown, along with their helicoidal arrangements determining polymorphs, and the semi-crystalline lamellae, bearing a bulk amorphous region consisting of amylose and disordered reducing ends of amylopectin around the hilum. Further growth rings depict amorphous regions of intermingled amylose chains and disordered, extended amylopectin side chains. The crystalline region encompasses crystalline and amorphous lamella and loose linear amylose strands over the granule.

Continuing with the structural examination, molecular patterns are also formed by a natural tendency of amylose and short chains of amylopectin to fold inward (see Figure 1), forming single and double helixes that crystallize, contributing to the differentiated crystalline regions that make up the starch granules’ semicrystalline nature by layering upon a common origin called the hilum [39]. If only one hilum is differentiated, the granule is deemed simple, but if several hila (differentiated by membranous cross walls termed septa) are present in tightly packed polygonal arrays, the granules will be called compound, and in some taxa, both types may be found, leading to a great assortment of profiles such as lenticular, polyhedral, or spherical granules, among others [40]. Particular starch biosynthetic pathways translate into a distinctive molecular architecture and ultimately express a species-specific starch morphology of simple or compound granules [41] that will impact the further technological or nutritional properties.

Granule size and sphericity have been negatively correlated with starch digestibility, and cereals usually exhibit higher digestibility in comparison to legume or tuber starches, as Corgneau et al. (2019) showed when studying conventional starches [42]. According to their reports, potato native granules with very high resistance to enzymatic hydrolysis had smooth surfaces without open pores, along with oval or round shapes, such as in maize and cassava, that also showed resistance; this is in contrast to rice, which, with its small, convex granules, is more rapidly digested. In addition, because it has a greater molecular surface area than amylose, amylopectin is expected to be readily digested. In their study, the aforementioned authors concluded that rice waxy starches performed as expected, but waxy maize, surprisingly, displayed a superior nutritional profile to its amylose-rich counterpart. These results highlight the importance of the botanical variety of the source, besides the storage, processing and domestic cooking conditions of the material; higher amylose rice starch hydrolysis is slower and less complete, for example [43]. In addition, environmental variability is important for rice amylose content, in part due to microclimates within close areas of rice growth, as nitrogen fertilization increases rice’s amylose content (an important quality trait), while elevated CO_2_, low light intensity, droughts, or high temperatures in the crop areas may decrease it in some cultivars [44].

As with most ingredients in the food industry, the highest purity is desired for starches. For a starch to be considered pure, it must consist of the stated carbohydrates in at least 98% of its total weight, with the rest of the minor components accounting for 1.5–2% of its weight: lipids, proteins, phosphates, fiber, and, in lesser amounts, bioactive compounds such as minerals, glycoproteins, or exopolysaccharides [45]. As indicated, different plant genotypes express unique molecular designs, where the botanical origin also influences minor components bound to the starch granule. Cereal starches, in general, tend to have lipids as their most common adjuncts, whereas starches from vegetative structures such as tubers or leaves show amounts deemed to be negligible. Due to the grain composition and amyloplast membranes being damaged in the extraction process, cereal starches exhibit approximately 0.5–1% of these compounds in the form of surface lipids, lysophospholipids, or free fatty acid molecules such as linoleic and palmitic acid [46]. The location of adjoints in the starch structure is also different depending on the specific crop and molecule. For example, oat granules may specifically bind lipids to amylose, but lysophospholipids and fatty acids in other cereals are found on the inner side of the granule; triglycerides, phospholipids, and free fatty acids predominate in the surface where proteins are present too, and hence, bind to membranes but can also reach internal cavities if they are not already present in bound or free forms [47]. Channels or pores in the granule surface surge from amylose/amylopectin nonreducing ends oriented towards the center of the granules connecting the central cavity to its surroundings. Pores are characteristically larger on type-A starches and less defined on B-type. Pores play an important role in the access of chemical reagents into the granule matrix, either by modulation through the multiple channels formed on the A-type or by blocking the few voids in B-type [48]. The pore structure constitutes important sites of catalytic reactions of digestion, an important fact related to the nutritional quality.

Other important minor components include intermediate materials such as polyglucans resulting from altered starch biosynthesis, besides elements such as phosphates, potassium, and magnesium. Phosphates, present in tubers as monoesters, as well as in an inorganic form, covalently bound to amylopectin or in association to lipids, are particularly relevant for technological purposes [47,49]. Some of the better elucidated molecular interactions between minor and major components in starch granules include covalent links of lipids and phosphates to C3 or C6 in glucose units, where amylose complexation entails free fatty acids occupying the inner nonpolar core of the single helixes in the molecule naturally, or as a consequence of gelatinization [41,46], with the technological outcomes therefore determined by the starch source and composition.

### 2.2. Nonconventional Hydrocolloids: Untapped Possibilities and Relevance

The presence of certain chemical compounds in plants has shown a high specificity for determined taxa, as happens with betalain pigments, exclusive to the Caryophyllales order [50]. However, hydrocolloids have been extracted throughout history from a multiplicity of clades, as reported by authors over the world, including American, Asian, African, and European plant species whose exudates, leaves, bark, seeds, or peels brim with substances ripe for food uses (Table 1). The key structural, physicochemical, and functional properties of many alternative HCs are yet to be studied, but currently available information on selected species is given in the following.

These hydrocolloids have a biological function according to the species, as part of cell walls, or intracellular fluids, but mucilage in different locations has particularly drawn attention lately. Underneath external coatings of fruits and seeds, pectinaceous polysaccharides are secreted by the Golgi apparatus of mucilage secretory cells, improving the development and survival of the structures through moisture increase under hydric stress, enhancing zoochory, or augmenting germination rates [89,90]. In addition to serving as nutrients, mucilages can set a barrier to water and oxygen, being surrounded by gel-like capsules upon imbibition. For the former reasons, mucilages have been harnessed by both the medical and food industries, focusing on species from the Brassicaceae, Solanaceae, Linaceae, and Plantaginaceae families [60]. Nonconventional hydrocolloids can even have provenances other than plant-based, as recently shown by extracted levans from microorganisms such as *Lactobacillus sanfranciscensis* and *Gluconobacter albidus*, which have proved useful for thickening edible fluid mixtures due to their natural structure as polydisperse fructose polymers [91,92]. Additional nonvegetable sources of uncommon hydrocolloids may be macroalgae, a renowned source of phycocolloids; they have also shown potential for compounds other than cellulose, Phaeophyceae alginates, and Rhodophyceae agar or carrageenans, through the discovery of a considerable number of important substances such as fucoidan, laminarin, and ulvan—chemically unique polysaccharides different from those of terrestrial organisms [93,94,95,96].

In terms of amylaceous hydrocolloids, it has already been mentioned how alternative sources of starches entail inherent structural differences in terms of the XRD patterns, granule size, rheological features, or nutritional value, but the possibilities these alternatives offer to food science and technology are yet to be placed in the foreground. For decades now, vegetable use for starch extraction worldwide has focused 75% on only one species, maize, with 24% contributed by remaining major crops such as wheat, cassava, potatoes, and, to a lesser extent, rice [22,97,98], bringing about a series of positive and negative associated consequences. In the first place, controlling some species allows for the standardization of processes, and regular outcomes for mass production—an advantage for industrial production. Nevertheless, large-scale monocrop landscapes are associated with biodiversity depletion, which leads to unsustainable agricultural systems and worries about food insecurity in the future [5]. In such a setting, a direct effect of the threat to biological systems welfare would result in major resources shortcomings, failing to meet not only the needs of food and other industries that rely on such raw materials, but further impact society around the globe, through the shutdown of commonly invisibilized ecosystem services [6]. Genetic pools in nature are the core of an agricultural system’s ability to thrive despite different crises, relying on natural plasticity to overcome new plagues, weather pressure, or diseases, to the point of being recognized as a public good, especially in the present challenging context [99,100].

In spite of being agriculturally recognized, simply because they are not major staples, some edible species are referred to as “orphan”, “neglected and underutilized species” or crops [101]. The research spotlight is turning to the urgent need to restore soil conditions and promote ecosystems’ resilience within the framework of current climate change [4,102]. This is a very important tool to reinforce the environment’s natural recovery ability to produce new resources later on, considering the challenges food-producing industries have in the face of a crisis that each day puts pressure on producers in the field on account of a growing population, erratic weather conditions, low mechanization, and market limitations during the current pandemic [103,104]. In contrast to modern genetically modified monocrop paradigms commonly found all over the world, wild edible species play a critical role in meeting rural families’ diversified food needs [7] while serving as in situ conservation reservoirs for natural resources, complementing germplasm banks and ex situ preservation alternatives [105]. Diverse systems have traditionally been a way to ensure a biological balance. Recent studies have challenged the traditional interpretation that pre-Hispanic people were as reliant as we are upon maize for subsistence, providing solid archaeological evidence of bone tools with leftover starch grains from the processing of potatoes, sweet potatoes, manioc, and *Oxalis* spp. tubers. This evidence suggests a greater role for diverse edible plants, especially, underground plant organs, in late pre-Hispanic subsistence [106], highlighting the importance of neglected and underutilized species. Furthermore, low-income countries without massive conventional starch species production, processing, or modification tools are forced not only to change their cultural food habits but to invest in the import of foreign species, leading to wobbling economies prone to constant crisis [107].

In light of these facts, considering today’s common staples as exclusive sources of starch is a questionable practice if aiming to ensure sustainable consumption worldwide in the long run. Therefore, possibilities to sidestep the issue should be studied, with countless options found in the literature as evidence of the natural resources available. Algae, for example, are already known for producing HCs and could have even higher biomass productivity than green plant crops, with starches with small granules (0.5–2.1 μm) that are useful in fat-replacing applications; as thick coatings for fabrics, high-quality biodegradable films, or paper; and as a carrier material for pharmaceutics or cosmetic/flavor essences [108]. Even residual biomass from different species from the *Closterium* and *Chlorella* genera, such as *C. vulgaris*, has proved useful in starch extraction, and for bioethanol creation [109]. If botanicals are preferred, when scrutinizing starch sources, photosynthetic organs such as leaves or reproductive structures are not regularly used for the industry as the amounts are variable and smaller than those of nonphotosynthetic seeds, unripe fruits, roots, rhizomes, bulbs, tubers, corms, and stems [41]. Several vegetable structures among these, from a multiplicity of sources around the globe, may constitute nonconventional alternatives to corn kernels, rice/wheat grains, potatoes, or cassava, coming in different shapes, sizes, and related applicability. Even species that grow in unsuitable soils for traditional crops and have not been recognized as having agricultural purposes may be interesting starch sources. For instance, okenia (*Okenia hypogaea)*, a wild plant from the *Bougainvillea* family in North America, has characteristic bright purple flowers that turn into starch-filled seeds; the starch they produce bears a strong resemblance to cornstarch and could be used in the production of weak viscoelastic gels with non-Newtonian behavior and thixotropic properties, along with a higher swelling capacity and a lower gelatinization temperature [110].

In addition to uncommon plant sources of different taxa, lesser-known or abandoned species commercially important in previous times are also an option, such as grass pea seeds (*Lathyrus sativus* L.), even used as famine foods in some wars and now common in Asia, Africa, and Southern Europe; summon 42–48% of their dry mass is high amylose, C-type starch of high purity, leading to high-viscosity pastes and stronger gels than other cereal-based starches [111]. Given of present-day conditions—dwindling resources and concerns about food waste—there is a determination to use fruit and vegetables that do not meet quality standards, such as stenospermocarpic fruits [112]. In the same way, large amounts of residues of seed, peel, pulp, or pomace fractions of less interest coming from purées, oils, and minimally processed vegetables leave the industry with potential grist for starch extraction [113]. Some of these are mentioned in Table 2, along with a series of native or endemic species pivotal to food sovereignty and security around the world. All of these have proven advantageous for the industry as starch sources, and offer the added perks of boosting local economies, being better for the environment, and contributing to a new academic body of knowledge.

To keep it simple, we have only mentioned a few lesser-known nonconventional sources of HC, but cutting-edge techniques where nanoparticles come into play have also been popular recently. In spite of not using alternative sources, an advantage of these techniques is the exploitation of byproducts from monocrop industries, e.g., cassava and potato peels, that have become part of circular economy solutions, through the development of starch nanoparticles aligning day-to-day engineering, science, and market aspirations [136,137]. Makroo et al. recognize a number of different uncommon fruits, seeds, beans, and byproducts as useful for the extraction of nonconventional starches, offering an alternative to “tediously produced conventional starches,” with advantages for industry also, such as decreasing the price associated with the process [138].

Starch may have different morphological and technological characteristics depending on the specific plant structure and geographic region of origin. According to the botanical source, a distinctive biosynthetic pathway is shaped into a unique molecular structure for subsequent amylose-rich/low starches with innate granules morphology, size, and technological properties for each HC species. Granule shapes, hence, range from round, oval, ellipsoidal, disk, polygonal, or truncated to irregular, with sizes going from some nanometers to more than a hundred micrometers, in species such as *Canna*. The composition of carbohydrate components in native starches generally showed the expected pattern, ranging from 14%, up to 56% in amylotypes, with high technological applicability associated with the continuity and firmness of gels, achieved by amylose as a cross-linking agent that increases intermolecular associations, in contrast with waxy starches that may exhibit rapid viscosity loss after paste formation [139]. Indeed, *C. cuspidate* starch, the highest amylose-containing starch of the sample, revealed high viscosities during and after testing, but the researchers highlighted how, despite the higher amylose content, lower gelatinization enthalpy was seen on the fruit starch, versus a similar starch from another local species, acorns. Lee et al. pointed to the relevance of the research on the crystalline structure within the granules to understand the differences in the gelation mechanisms of both Fagaceae starch sources [134].

Most countries have an assortment of biological potentialities according to the climate, relief, and corresponding ecosystems. Due to human intervention, species have spread far from their origins, increasing even more the available diversity of supplies, with a sustainable approach that encompasses ecological considerations for the system to be sustainable. A feature learned over time, for example, has taught technologists to extract starch from nonphotosynthetic tissues such as those pictured above, where starch is stored, instead of facing the constant daily turnover that transitory starch goes through [41]. Many native and underutilized species may carry a disadvantage for the industry because of their limited harvest, sometimes acquiring one or two picks a year maximum. However, more than 30,000 species have been cataloged as edible over the world [140], with many of them harnessing HC as intrinsic components, and that is only in the plant kingdom, so an array of possibilities is still available in different seasons to exploit natural resources responsibly, and most of them have yet to be explored for food applications.

## 3. Technological Considerations

### 3.1. Hydrocolloid Extraction and Processing Technologies

The raw vegetable matter, HC chemical structure, and affinity for polar or nonpolar solvents determine the extraction method utilized for the specific hydrocolloid of interest. Regularly, physical processes are utilized to extract gums, by drying, grinding, and mixing the plant tissues with water, ethanol, or oils, then centrifuging, evaporating, and refining the powdered end products. Based on the operation conditions, the extraction yield, degree of purity of the compound, and characteristics of the HC will vary. High-temperature drying of grains for starch extraction, for example, is used to increase the rate of the operation; however, it triggers high-stress cracking, low resistance to mechanical impact, low starch recovery, steepwater solid loss, poor starch paste viscosity, increased gelatinization temperatures, decreased gelatinization enthalpy for regular cornstarch, and additional water input being needed for gelation when treating with waxy genotypes [141].

In terms of starch isolation, the osmotically inert and poorly water soluble nature of starch is a central characteristic to bear in mind during the process. When cereals are the raw matter, dry and wet milling are performed as means of extraction, with the latter preferred because of its higher efficiency [142]. The processing parameters of the starch obtention operations are very important, since the technical performance of the starch is limited by certain conditions. In the case of grain wet milling, the first step involves steeping grains with reagents such as metabisulfite, lactic acid, and sodium hydroxide to facilitate protein release, followed by milling, screening, and centrifuging procedures to eliminate remaining proteins, lipids, fiber, or water-soluble materials. In contrast, for vegetative organs such as stems, tubers, or roots, the grinding or shredding of peeled, clean materials is carried out until a slurry is formed that will later be screened and centrifuged [22]. Instead of initiating the process with raw plant structures, isolation may also be performed using flours as dry method milling states, before aqueous extraction, and wet milling is followed either by hydrocyclone separation or the “tabling” method, where density differences of starch versus protein are put to use in a table where a slurry is inclined, separating the compounds [142]. In general, the most common methods make the starch available through hydration and downsizing the particle size of materials.

Innovative technologies have been added enzymatic, microwave, or ultrasound assistance in order to increase the HC extraction yield, lower the energy input, shorten operation time, and/or reduce the use of solvents, consequently ameliorating operators’ safety, diminishing extraction risk, and improving extract quality [56,143,144,145,146] as described below.

#### 3.1.1. Enzymatic Extraction

Enzymes are utilized because of their ability to hydrolyze tissues to make HC available, and the concomitant release of the molecules’ inner linkages, eliminating sulfates, prompts polysaccharide separation, and induces the epimerization of acid residues in agar, alginates, and λ-, κ-, and ι-carrageenans [147]. As a result, polysaccharides’ hydrolysis into smaller dimers or trimers has an effect on the gelatinization and solubility properties, and Rhein-Knudsen et al. [147] highlighted that agar gels with a lower content of sulfate anions have increased strength, a higher melting point, and lower viscosity at 60 °C in comparison with carrageenan. Additionally, the presence of hydrophilic sulfates determines the water solubility of carrageenan, lowering the solubility temperature and, if anhydrous bridges are present, as a consequence of sulfate esters’ elimination, the viscosity may likewise be distorted. Carrageenase reactions must be controlled, taking advantage of their specificity, as desulfation may cause the phycocolloid to lose gelling properties. Attention has similarly been given to the effect of enzymes in alginates, whose gel forming, water binding and immunogenic properties rely on the relative amount and sequence of M- and G-residues formed by epimerization reactions; this is an opportunity for the customization of specialized technological properties [147].

#### 3.1.2. Ultrasound-Assisted Extraction

This technology delivers fewer impurities and an increase in the antioxidant activity, lightness, emulsifying properties, and extraction yields of mucilaginous HC [56]. Just 20 s of the treatment proved to be enough for extracting the totality of the *Arabidopsis* seed mucilage. This improvement was a consequence of the cavitation phenomenon due to ultrasound wave diffusion, which produces turbulence that facilitates deisolation through microscale shearing forces and mechanical shocks of collapsing bubbles that ease solvent diffusion through membranes and mass transfer during extraction. As the temperature, time, and sonication intensity increase, yields increase proportionally, up to a certain limit; however, the first parameter is considered more significant, along with the proportion of solvent to raw matter, and the pH for products such as psyllium [56].

#### 3.1.3. Microwave-Assisted Extraction

This technique has gradually been introduced in laboratories as well as to industry for drying foods and enzyme/microorganism inactivation, and as assistance for extraction methods [145]. The technology involves radiation waves, of frequencies from 300 MHz to 300 GHz, that encounter polar compounds on cells, producing internal heat through ionic conduction and dipole rotation, rapidly inducing tissue damage and solvent diffusion. Cocoa husks, sugar beet pulp, and citrus, dragon fruit, and papaya peels are byproducts with a significant amount of pectin that, with the modulation of the solvent ratio, time of exposure, and microwave power, yield considerable amounts of HC, making them of great interest for the food industry.

### 3.2. Minor Components’ and Other Elements’ Effects

After the extraction, with or without assistance, hydrocolloids with the highest purity possible must be generated. If the task is not fulfilled, one of the main concerns for the industry is the remaining minor components of ash, lipid, or protein in foodstuff formulations.

**Figure 2:**
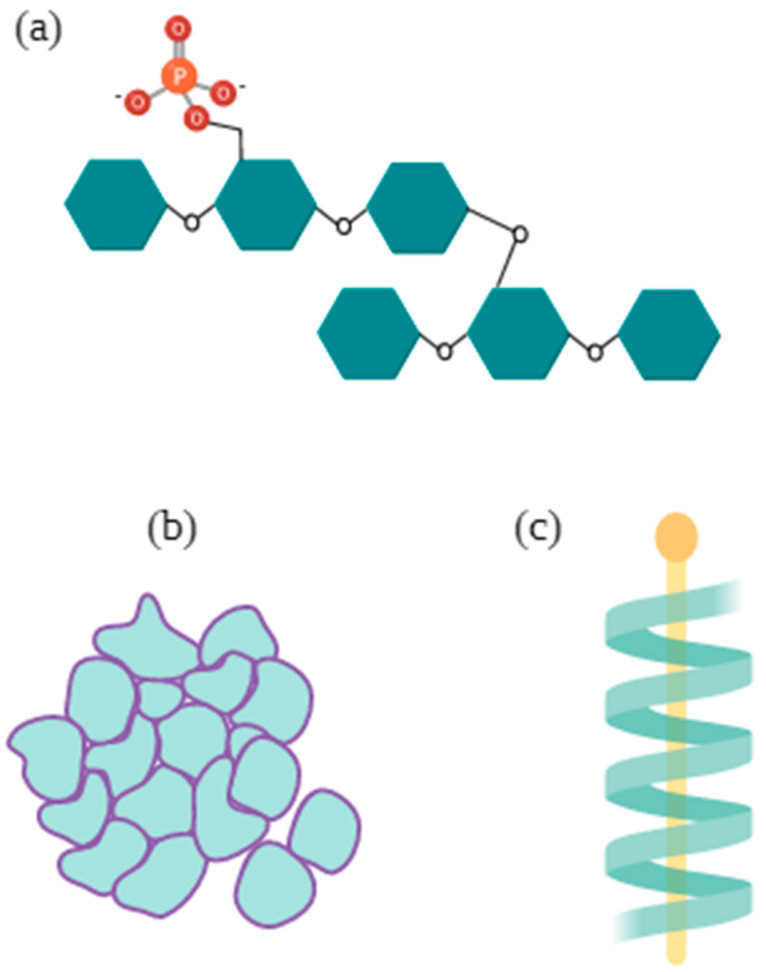
Minor components’ interaction with starch. (**a**) Phosphates bound to C3 or C6 glucose residues portrayed in cyan of amylopectin lateral chains; (**b**) protein matrix around starch granules; (**c**) fatty acid complex with amylose or lateral amylopectin chain helix. Based its use on [47,148,149].

Starch technological performance is closely related to its composition, as, although in proportions of less than 1%, nonamylaceous components such as glucose-bound phosphate residues (see Figure 2.) determine several traits. For example, starch granule hydration and lower crystallinity induce starch pastes with higher transparency, viscosity, and freeze–thaw stability [41].

Similarly, because of the processing effects on the starch depolymerization, it is very common for the fatty acids left in the starch to form stable complexes with the carbohydrate by penetrating amylose or amylopectin end helices (Figure 2). Either by surface interactions, inner monoacylglycerol meddling, or complexation with lipoproteins, lipids may have an impact on granule swelling by preventing amylose leaching or changing the gelatinization temperature or peak viscosity. This might help improve the textural properties of bread, including lowering the staling rate [148]. Disadvantages to the residual presence of lipids or proteins, namely an impure starch, include the high reactivity of the macronutrients to starch physical, chemical, or biological modification techniques [47], where reactions may involve the carbohydrate or influence:

- Formation of toxic compounds
- Generation of free radicals
- Maillard reactions
- Impairing of amylolytic enzymes
- Changes in protein crosslinking.

Additionally, the technological performance in terms of gelling purposes may be affected by the presence of ions with positive or negative charge changing a water system’s structure. Commercial starches such as cassava, maize, and potato mixtures have resulted in viscosity parameters of intermediate magnitudes, and aqueous chloride, sulfate, and sodium hydrogen phosphates have shown different effects, with sulfate having the most dramatic effect in terms of lowering the peak and setback viscosities [122]. The influence of KCl on C-type *A. fortune* gels, already causing two gelatinization peaks before salt addition, showed a pronounced increase in gelatinization temperature, contrary to A-type rice starch and B-type potato starch, which, irrespective of the KCl concentration, showed a single gelatinization peak [128]. Proso millet (obtained from *Panicum milaceum*) has similar behavior, with NaCl increasing the gelatinization temperature of the starch, but Zhang et al. noted the effect of thermal and pasting properties due to changes in water activity induced by salt and pH—in comparison to other waxy starches, proso millet was stable in a wide pH range from 4 to 10 [125].

The main drivers to integrate HCs in a food formulation usually involve rheological or structural reasons, with mostly but not exclusively edible purposes—making use of them as emulsifying, film-forming, binding, clouding, crystallization-inhibiting, clarifying, molding, suspending, swelling, color-enhancing, encapsulating, or fiber--adding agents [10]. Food processing industries frequently rely on a combination of controlling thermal treatments, biocides, pH, and water contents to ensure microbiological safety, a task that includes not only unitary operations but also the addition of ingredients to fulfill the purpose that may also influence textural properties. Two of the most renowned roles of the HCs when modulating vegetable and animal-sourced foods’ texture are thickening or gelling; however, the effect of electrolyte content, pH, soluble solids, and temperature must be kept in mind to ensure the HC accomplishment or stability. Saha and Bhattacharya [17] have noted how electrolytes may reduce the high viscosities achieved by carboxymethyl cellulose without affecting other cellulose derivatives or gums, and how many commercial gelling agents can build thermally reversible gels depending on the pH or sugar content, as pectins do. Traditionally, food applications consider the specificity of HCs’ requirements to perform, as cellulose that depends on electrolytes and pH for its use on sauces; pectins relying on high acidity, and either high solid dispersion for high methoxyl pectins to form thermo-irreversible gels when cooling jellies, or metallic salts for low methoxyl pectins to form thermo-reversible gels of jams [17]. Their molecular framework explains this phenomenon, as several HCs have an electrolytic nature. For example, chitosan is a polysaccharide with a cationic amino group, while carrageenan or alginates are typically anionic compounds and, due to these characteristics, they are known, along with other HCs, namely gum arabic or pectin, as polyelectrolytes [9,11].

Other nonamylaceous HCs such as Persian gum have also shown the effect of minor components in its structure, with protein and lipids possibly contributing to the exudate emulsifying activity [84]. Still, the spotlight is on the influence of soluble solids, acidity, and salts on these HCs. In phycocolloid systems, the presence of potentially associated ions is very important, as sodium, calcium, magnesium, and potassium, especially for κ-carrageenan, promote the cation-dependent aggregation of carrageenan helices for stiff gels to form by binding negatively charged sulfate groups, thus stabilizing helicoidal junction zones [147]. *Plantago* gums’ viscoelastic properties are also affected by the addition of sodium or calcium chloride, as strain values increase in direct proportion to the salt concentration, with Ca^2+^ cations being more effective than Na^+^ at strengthening the firmness of the gels [15]. The mineral content’s contribution to the viscosity of starches has been stated, and quinoa starches have been shown to naturally contain Ca, K, P, Mg, and S, affecting the physicochemical properties, acting as a thickener, and adding nutritional value due to its mineral-containing formulations [115].

### 3.3. Hydrocolloid Modification

On top of the molecular interactions of the aqueous media with intrinsic HC composition, consideration must be given to the physical conditions in which the reactions are conducted. Many HCs in their native state do not react readily with water at room temperature or regular atmospheric pressure. Therefore, interventions in their chemical structures are carried out to broaden their implementability in the food industry. Cellulose derivatives and starches top the list of modified HCs. Starches have been the main subject of modifications, being exploited in a multiplicity of applications that transcend alimentary uses [108].

**Figure 3:**
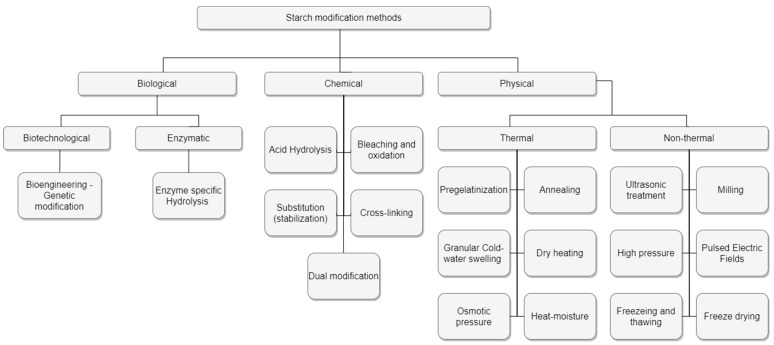
Modification methods for starch. Based on the methods of starch modification reported by [45].

Modification of HC may be achieved by physical, chemical, and biological methodologies. Massive starch sources have been one of the main targets of the related research [150], as modified starches have been broadly stated to be “superior materials” in contrast to their native counterparts in terms of the improved characteristics of hydrogels [151]. An assortment of techniques has been used for the modification of the HCs through the exploitation of available technologies, including bioengineering options (Figure 3).

Among the modification methods, the chemical approach, implemented since the first decades of the last century, has helped to overcome native starches’ water insolubility and retrogradation ease through the insertion of a new functional group on the starch backbone [150]. The structure modification requires the action of some reagents, sparking safety concerns about the technique. Toxicological regulations on the introduction of substituent groups have been developed, allowing maximum levels of acetates, phosphates, and hydroxypropyl of 2.5%, 0.4%, and 10%, respectively. Cross-linking modifications of one substituent group per 1000 or more anhydroglucose units are considered safe, with legislative approval given to the use of novel starch derivatives in processed food formulations [22].

Bioengineering efforts are being made to design species with higher starch yields or starches with specific amylose–amylopectin ratios. It is still challenging to determine the specific pathways involved in starch biosynthesis, structure phosphorylation, granule size, and maintaining structure, while the carbon flow is led towards its fixation. Inhibiting starch degradation is an important way to enhance deposition, as well as inducing heat resistance in upcoming climate change scenario, and specific amylose and amylopectin chains manipulation are also being assessed [45]. Professor Andreas Blennow, in Sjöö and Nilsson [45] highlighted how promising are the developments, aiming to decrease some starches’ percentage of amylose, and the main fraction linking lipids; decrease gelatinization viscosity, slowing down retrogradation, especially in cereal starches; or modify minor components bound, as smaller amounts of protein could mean diminished unwanted odor, color, easing of enzyme attacks, and hydration. They also explained how enzymatic techniques, with action mechanisms including the catalysis of starch hydrolysis, debranching, and transfer reactions producing new α (1–6) links, increase nonconventional starches’ nutritional value.

In addition to amylaceous HCs, phycocolloids have been widely modified by carrageenases, sulfatases, sulfurylases, agarases, lyases, and epimerases to improve the gelation properties of native HCs [147]. After gelatin, the second most common stabilizer of ice creams is kappa carrageenan, possibly due to its interaction with ice crystals’ surface in a similar way to antifreeze proteins, a property that is highly strengthened by k-carrageenan hydrolysates and has been taken advantage of through β-galactosidase hydrolysis [152]. Kamińska-Dwórznicka et al. compared the recrystallization of ice in 50% sucrose solutions with the addition of 0.01% k-carrageenan, 0.005% hydrolysates after 48 or 72 h (the time required for the molecular weight to decrease), and found an important retardation in ice recrystallization, especially strong with the longest enzymatic break decomposition time [152].

Physical modification methods are often preferred because of their lower cost, ease of implementation, low reagent residuality, and environmental safety. New technologies are emerging using radiofrequencies and instantaneous controlled pressure drops combined with pressurized steam, gamma, electron beam and ultraviolet radiation, and nanotechnology [45,150,153] as pictured in next paragraphs.

Grafting, the covalent attaching of chemical moieties to a polymer’s backbone, is achieved by various routes, one way utilizes radiation energy from photoirradiation, microwaves, infrared, gamma radiation, or electron beams to generate reactive species that bind added monomers, and have already been tested to depolymerize guar galactomannan, polymerize various monomers, or phosphorylate starch [16].

Extrusion may use thermal treatments in the presence of water, that induces conformational changes in gums, removing undesirable flavor and enhancing the water solubility and hydration capacity of fenugreek seed gum [63]. In nonconventional flours with high starch content, such as green banana, Giraldo et al. modified the XRD pattern of native starch by submitting it to extrusion, with the resulting amorphous material losing the typical morphology and modifying the thermal properties to increase the powder’s solubility, favoring the development of a fortified banana drink [154]. Structural modifications on alginates’ native structures, consisting of lineal β-d-mannuronic acid (M) and α-l-guluronic acid (G) linked by (1–4) glycosidic bonds, show that an increase in G guluronic acid gives HCs the ability to produce gels with higher firmness, transparency, and brittleness [11].

Supercritical water has also been used recently to modify *Cucurbita moschata* polysaccharides, using temperatures ranging from 120 to 210 °C and a pressure of 10 MPa [83]. As a result, the molecular weight distribution changed, the viscosity decreased, and the activation energy and flow behavior indices increased. Heating at 150 °C led to the highest values of antioxidant and emulsifying activity, improving the hydrocolloids’ overall solution properties and possible bioactivity.

Modified *Lepidium perfoliatum* seed gum, through cutting-edge cold plasma treatments at atmospheric pressure, produced a comparable performance to commercial xanthan or locust gums. The composition of this gum showed higher numbers of carbonyl and carboxyl groups, and increased water solubility for the production of stronger intertwined networks [61].

The esterification of basil seed gum through a common method for starch modification made it into a high biocompatibility, low-cost, edible HC thickener. The esterification reaction introduced octenyl succinic anhydride (OSA) into the gum structure of basil seed extract, improving its performance on water-resistant films’ smooth surface and enhancing its barrier properties [155].

Individual techniques led to improved thermal properties and reduced melting enthalpy through cross-linking, while the opposite effect was seen for oxidation, recalling the relevance of modification methods on specific HC sources. For example, nonconventional HCs such as sesbania seed gum underwent cross-linking and oxidation methods to yield a gum with affected swelling power, freeze–thaw stability, and viscosity [156].

### 3.4. HC Interactions

Food design often makes use of HC mixtures to improve the sensory and technological characteristics of products, as their performance is usually improved when put together. Gelling and nongelling HCs are combined to improve or induce gelation in food systems, as happens with xanthan gum and galactomannans that acquire a gelling action, usually absent or added individually, due to the synergistic effect between them [17]. Such interaction is one of the most extensively and longest studied in synergistic gelling systems, and alternative sources of HCs, such as tropical gums from Africa, are being assessed in combination with regular ones. Bocco, Khan, Kelly, Goudo, Lalo, Mango, and Nkui gum extracts (from leaves, nuts, barks, almonds, and shoots) showed a positive interaction with alginate in the presence of calcium chloride. The latter compound enhanced synergy as it reduced the electrostatic repulsions inherent to gums’ negative charge, and allowed for newly acquired gelling properties, besides thickening capabilities [79]. Besides, the former authors emphasize the relevance of the intermolecular interaction in terms of electrical charges that restrain the synergy between alginates and pectins, gelling only at low pH where protonation of the carboxyl group is given—a phenomenon otherwise impossible at neutral pH, where gums face electrostatic repulsions between them.

When analyzing the technological performance, a great hindrance in native starches being used for industrial applications is their susceptibility to retrogradation, ordinarily related to the high amylose content. One way to amend this without reforming their structure is to blend them with other nonstarch HCs such as pectin, arabic, guar, gellan, locust, or xanthan gums for a synergistic interaction that widens their suitability [157,158].

Huang et al. [159] found that mucilaginous HCs’ addition to tuber starches affected the rheological behavior of native yam, taro, and sweet potato starches, causing a noticeable increase in the onset temperature while decreasing the phase transition temperature range (Tc–To) and enthalpy (DH). The addition of mucilage also resulted in a slight decrease in swelling power for yam and taro starches, but did not change that of sweet potato starch. The results suggest that the effects of mucilage on gelatinization and pasting properties are reliant on the starch source and the concentration of the mucilage.

According to Zhang et al. [126], an HC blend system with starch and nonamylaceous water colloids may be achieved in three ways: mixing starch/HC powders into the water, adding starch to a prepared HC solution, or making a complex viscous mixture system by mixing the prepared HC solution into a starch paste [160]. These, however, cause only superficial interactions, but Zhang et al. proved how, through methods such as partial gelatinization and subsequent freezing/thawing, xanthan gum attaches to the surface of potato starch when the soluble starch is rearranged into a stiffer matrix. This combination of methodologies improved the thickness of the semicrystalline lamellae and the shear stability during pasting, useful for many food applications.

HC blends may even have an effect on their modification processes: it has been shown that xanthan gum, added to corn, potato, and tapioca starch, enhanced the effect of physical heat, freezing–thawing, and combined treatments, stabilizing gelation phases particularly for the nightshade tuber [161]. Dry-heated tigernut (*Cyperus esculentus*), a nonconventional starch, was effectively modified with another alternative source of HC, Chinese quince (*Chaenomeles sinensis*) gum. The modification resulted in a protective role of the latter that allows for protection of the starch granules’ surface and crystalline structure, which are otherwise destroyed by thermal effects; additional benefits of the starch are increased viscosity and freeze–thaw stability [162]. Comparably, chestnut starch thermal properties were decreased by dry-heating, but when xanthan gum was added, the gel structure was uniformized, with the added benefit of an increase in health-promoting resistant starch [163].

## 4. Nutritional and Biofunctional Considerations

### 4.1. Bioactive Compound Protection

Functional foods are composed of biologically and physically active (bioactive) compounds that provide health benefits beyond their nutritional value; from animal, microbial, fungi, algae, or plant origin, these impact health through their prevention of cardiovascular disease and type II diabetes and their antioxidant, anticancer, and antimicrobial activity [164].

Some HCs are used for protecting foodstuffs from decay by engineering edible or biodegradable barriers to dehydration, physical, or potential microorganism harm. Water-soluble compounds may be lost through dehydration, but many bioactive substances are also prone to degradation because of exposure to light, oxygen, or processing. At a molecular level, HCs have been used as micro- or even nano-encapsulant agents, conferring controlled release, high bioavailability, and stability to formulations with bioactive substances that promote health [28]. Other systems guarding bioactive compounds against deleterious effects by HCs are emulsions, biopolymers, nanoparticles, and microgels [165]. On a larger scale, HCs such as okenia, banana, and mango starches have been successfully incorporated in films and shown acceptable physicochemical, mechanical, and barrier properties; thinner films can be derived from okenia as a result of its low amylose content [166]. Similarly, lentil, pumpkin, and quinoa starches have shown higher water vapor permeability in comparison to potato starch; meanwhile, the swelling capacity of lentils was the lowest, opposite to pumpkin and quinoa [167].

### 4.2. Bioactive Role of HCs

The potential of hydrocolloids as health promoters per se has been widely recognized [28,158,165], and recently both technological and bioactive roles have been considered in food formulations to fulfill consumers’ needs and world health goals. For example, some unexploited HCs such as Iran’s Ang gum, with verified effects on rheology and inducing a superior performance on emulsion stability versus commercial gums such as Arabic gum, have traditionally been used for medicinal purposes [168].

The categorization of bioactive compounds considers effects on human physiology. One of the most discussed effects in relation to starch consumption is its effect as dietary fiber. Starch submitted to enzyme action in vitro is classified as rapidly digestible starch (RDS), slowly digestible starch (SDS), or resistant starch (RS). RS is of major interest for health issues, as it is a source of fermentable carbohydrates for colonic microflora, being associated with prevention of inflammatory bowel disease, restoration of apoptosis, and reduced incidence of colorectal cancer, besides management of obesity, glycemic responses, and the blood lipid profile [153]. High RS is desirable in foods from a nutritional point of view, and from a technical point of view it has been reported to improve crispness, expansion, mouthfeel, color, and flavor, but processing usually decreases its amounts through variations in pH, high temperatures, and handling time, besides cooling cycles, freezing, and drying [169]. Nonconventional starches such as *Coix lachryma-jobi* cereals have been successfully modified through enzymatic means, giving them a higher nutritional value compared to pregelatinized native starches [45]. As mentioned, some nonconventional sources of starch provide RS after modifications, such as chestnut [163], but native starches from nonconventional sources may also be an RS source, as suggested by studies on the African yam bean [170], edible canna [124], or ahipa [129]. In whole foods such as pulses, the low postprandial glycemic response is associated with a diminished risk of obesity, diabetes, or cardiovascular disease, probably related to the water-insoluble protein matrix. HCs, when added to amylase inhibitors such as tannins, phytic acid, or lectins present in such legumes, contribute to a lower starch digestion rate [171].

For some authors, the health value of hydrocolloids is limited to the regulation of physical effects (gastric emptying time, intestinal nutrient passage rate, nutrient absorption, and digestion) or the molecular and cellular effects induced [28]. As to the former, many HCs have proved their role in the modulation of macronutrient digestion and absorption, possibly promoting nutraceutical or vitamin bioavailability, reducing fat/starch uptake, or promoting a healthy gut microbiome through their prebiotic potential [165], besides the previously named benefits. Namely, because lipases have to trespass the gel network of HCs, digestion rates are reduced for fats, as lipid droplets are encapsulated by the hydrogel matrices formed, for example, by alginates binding calcium ions to avoid long-chain fatty acid precipitation during digestion [165].

Some consumers may not be familiar with some of the names of HCs and associate chemical compounds’ names with potential health threats, thereby decreasing the acceptability of this type of strategy [172]. Moreover, some studies have indeed questioned the safety of some HCs’ long-term effects, such as carrageenan, whose effect in predisposed populations is not clear and has been associated with compromised human health and well-being [173]. Physical technologies are then preferable for HC obtention or modification, and local sources that may be familiar to people are preferred because familiarity may improve consumers’ perception. Certain HC byproducts from processing or digestion may have negative side effects, as happens with carrageenan, such that it has been discredited and is avoided by consumers and manufacturers, in consideration of dangerous hydrolysates. An example of such a molecule is poligeenan, a fraction of the phycocolloid derived from carrageenan intense heating or acid hydrolysis, causing intestinal ulceration, ulcerative colitis, or Crohn’s disease in animal models [173]. Then, there needs to be greater attention to the algal substance processing to prevent the appearance of these kind of compounds with molecular weights less than 100 kDa.

### 4.3. Special Considerations in Health Conditions

A recently studied health condition induced by foods is coeliac disease, an immune condition inducing small intestine inflammation, which has an increasing prevalence around the world [174]. Numerous industries rely on HCs, and recent trends associated with coeliac disease are driving the demand for more HCs for the development of new products, replacing traditionally cereal-based, gluten-rich choices [174]. Recent uses of starch in the food industry underline the characteristics imparted to the product. It aids the substitution of fats in dairy products or mayonnaises, improves the texture of baked goods or snacks, structuring high-temperature processed foods, and is useful when powdering marshmallows. It can also be used for sweetening or, because of its nutritional contribution, as fiber in baking, and for the encapsulation of bioactive compounds such as pigments or flavor [45].

Although the main biofunctional roles recognized for HCs include: the amelioration of constipation by *Plantago ovate*, the prebiotic effect of inulin, or cereal β-glucans hypoglycemic effect [175], some of the many HCs documented in this review are still to be tested for bioactivity. In general, prebiotic effect is also related to the effect of dietary fiber on health in terms of a lower cardiovascular disease risk, gut health, diabetes or glycemic control, reduced risk of cancer, improved immune function, lower body weight, and improved bioavailability and calcium uptake [176]. Natural locust, guar, tragacanth, and arabic gums have been proven to be effectively prebiotic for bifidobacterial synbiotic applications on animal systems [177], depending on the host diet for the carbohydrate utilization. Different starches that resist digestion through the gastrointestinal tract have shown potential as functional ingredients or components of dietary fibers [178]: chestnut [163], banana [129], ahipa [126], *canna* [124], stenospermocarpic mango [112], or *D. roosii* [131] starches may be sources of resistant starch, improving health.

## 5. Future Considerations and Prospects

When large investments are involved on an industrial scale, cost-effective raw materials, efficient equipment, and long-lasting technologies are preferred, chosen with aspects such as market dynamics and product availability in mind. Consumers drive the market-set boundaries for these considerations, and a demographic shift has been recognized recently, associated with the increase in the elderly population around the world. When this factor is added to food design priorities, it must be remembered that the elderly may look for different products, including softer, semisolid foods due to chewing difficulties or dysphagia; industry can meet this demand by 3D printing restructured vegetables [179,180]. In conjunction, plant-based diets keep growing in popularity due to ethical, environmental, safety, and health concerns such as zoonotic diseases [179,181]. Therefore, vegetable-origin high-protein foods have been designed, without sensory compromise, thanks to the use of HCs.

In addition to industrialized trends, in the last few decades, an alliance has propagated over the world, bringing together chefs, gastronomists, and natural and social scientists to increase food knowledge from a joint perspective, giving rise to concepts such as gastrophysics or gastrolabs, and bringing about scientific views on nutritional and sensory concerns involved in the design of new dishes [182]. Chickpea, common bean, Peruvian carrot, sweet potato, and white bean starches, for example, have demonstrated high performance in pound cakes, in contrast to commercial cornstarch, increasing the specific volume, lightening the color, and improving the texture and crumb moisture during storage [183]. Molecular gastronomy has particularly taken advantage of phycocolloids through techniques such as spherification or gelation to form amaretto fondants, ice cream envelopes, arugula spaghetti, or caviar from alginate, celluloses, and agar, for example [184]. Nonconventional HCs are also used in cooking, with soybean polysaccharides, tamarind xyloglucan, cereals β-glucans, microbial alternan, elsinan, scleroglucan, or pullulan being used to, e.g., prevent retrogradation in sponge cakes, increase stability in frozen pasta, make gluten-free bread, decrease the fishy smell of preserved mackerel, or improve the texture of Basque omelets and cookies [185].

## 6. Conclusions

A variety of novel hydrocolloids from different species besides those already mentioned, such as sage, Balangu—*Lallemantia royleana*, Qodume shirazi—*Alyssum homolocarpum*, Espina Corona—*Gleditsia amorphoides*, Qodume Shahri—*Lepidium perfoliatum,* cashew—*Anacardum occidentale* seed gums*,* Chubak—*Acanthophyllum glandulosum* root gums, marshmallow—*Althaea officinalis* flower gum, or *Opuntia ficus indica* mucilage [10] are yet to be explored, modified, and exploited. A fraction of the species around the world is mentioned in this review, as a sample of the huge potential of this topic.

Nonconventional starches from alternative sources are now under scrutiny by both scientists and technologists around the world to meet contemporary demands and socioeconomic and environmental challenges. Exudates or extracts from traditionally forgotten algae biomass, or plant species and structures, are flourishing in light of the need for agro-industrial food security and sovereignty.

Different taxonomical origins for HCs are shown: depending on the geography, the specific biotic and abiotic conditions determine the vegetable species available and its composition in terms of useful phytochemicals. Of the listed taxa, botanical families conventionally recognized for HCs extraction such as Fabaceae (from which tragacanth, guar, and locust bean gums are obtained) and Malvaceae (from which karaya gum is isolated) also appear, with the different genera and species exemplifying the unexplored potential of the world’s flora. Some of the seeds, but also the barks, fruits, and leaves, of plants from different ecosystems and continents have found medicinal, ornamental, or food uses in the past, but have also recently proved to be HC-rich, having been mentioned as natural sources of innovative hydrocolloids with the ability to modify water-based foods.

New technologies arise from the discovery or recovery of many byproducts or neglected and underutilized species, whose potential might be seized upon to improve the efficiency of current HC harnessing.

Food HCs play an important role in foodstuffs as modulators of molecular interactions between water-based systems. Being among the most common and advantageous HCs, gums and starches are used daily in different industries considering their biological origin, interaction with other molecules, and the extraction or modification operations to which they are subjected. Taking advantage of knowledge on HCs’ composition, food scientists and technologists have improved the extraction yield through the assistance of biological, chemical, or physical methods such as enzymes, ultrasound, extrusion, or microwave. HC mixtures have also served to improve overall products performance, either at the food formulation stages when synergic effects are reported, or by protecting bioactive molecules, and enhancing modification processes, as happens with alternative starches and traditional HCs such as xanthan gum. The gradual incorporation of some of these nonconventional sources of HC, taking advantage of natural characteristics (that may lower operational costs) and considering both the diversity and harvesting cycles of different botanical sources, is strongly advised to meet world food needs.

Health considerations are also mentioned, such as the decision to no longer use some traditional HCs because of the risk they entail (such as carrageenan and low-molecular-weight hydrolysates), but also, e.g., the possibility of improving both physical and mental health through the addition of fiber to the diet, the prevention of noncommunicable diseases, the improvement of the sensory qualities of gluten-free baked goods, and restructuring products for an increasingly elderly world population. Novel sources of HCs may be assets in terms of the current context of genetic erosion, promoting better food systems in the face of today’s and future global challenges.

## Figures and Tables

**Table 1:** Botanical origin, compositional, and functional properties of some nonconventional hydrocolloids.

Botanical Family	Species	Common Name ^1^	Plant Structure	Extraction Yield	Molecular Weight	Composition	Rheological Behavior	Additional Remarks	References
Fabaceae	*Cercidium praecox*	Brea	seeds	76%	122 kDa	75% carbohydrates, 9% proteins, 2.6% acetyl, 17% uronic acids	Pseudoplastic behavior with viscosity ≈ 110 Pa·s	Brea gum decreases corn oil droplets and polydispersity in emulsions, while increasing apparent viscosity and stability in a better way than gum arabic.	[51,52]
Brassicaceae	*Lepidium sativum*	Garden cress		8.97 ± 0.12%	1090 ± 8 kDa	Predominant monosaccharides: rhamnose 11.87 ± 0.41, arabinose 11.02 ± 0.16, xylose 9.06 ± 0.18	“Non-Newtonian behavior with complex viscosity (At ʄ = 1 Hz) = 3.078 ± 0.015 Pa·s	Cress seed gum reduces native wheat starch gel retrogradation, gel hardness and syneresis.	[53,54,55]
Brassicaceae	*Camelina* spp.	Camelina		19.08%		75.1% polysaccharides and 12.3% protein	Newtonian flow behavior at low shear rate (less than 0.011 s^−1^) with a viscosity of 62.80 Pa·s, with a concentration of 1%, it reaches 350 Pa·s, higher than k-carrageenan 100 Pa·s and HEC 80 Pa·s	Camelina gum serves as a food stabilizer, emulsifier, and gelling agent with higher viscosity, storage, and viscous moduli than conventional components.	[56,57,58]
	*Alyssum homolocarpum*	Qodume Shirazi		9.87%	122.5 × 10^6^ g mol^−1^	Anionic rhamnogalactan consisting of 61% carbohydrates, 17.9% proteins, and 10.9% uronic acids	Pseudoplastic behavior with zero shear viscosity = 19.24 Pa· s and infinite shear viscosity of 0.0013 Pa· s when the concentration is 1% tested at 25 °C.	The multiple negatively charged carboxyl groups on *A. homolocarpum* gum, along with its the low sensitivity temperature changes in dispersions, makes it into a useful stabilizer for foods requiring thermal processing.	[56,59]
	*Arabidpsis thaliana*	Mouse ear cress		Increased up to 6-fold in ultrasonic assisted extraction versus chemical agents.		Primarily rhamnogalacturonan I, besides of cellulose, galactan, xylan, arabinan, and homogalacturonan		*Arabidopsis* mucilage comprises a water-soluble, nonadherent external layer, and an internal adherent one with similar sugars to commercial HCs, increasing its potential as a food thickener	[56,60]
	*Lepidium perfolatum*	Clasping pepperweed		17.63%	200 g/mol	Protein content of 2.84%, color hue angle coordinate 60.5°, and emulsion stability of 88.96%	Non-Newtonian behavior with viscosity = 463.07 mPa·s and peak at 519.8 mPa when extracted at 45 °C for 1.5 h at pH 8.	*L. perfolatum* shows great potential for commercial competitiveness, as its extraction yield is greater than other nonconventional gums such as yanan, flaxseed, malva nut, mesquite seed, fenugreek, or Opuntia mucilage	[56,61,62]
Fabaceae	*Trigonella foenum-graecum*	Fenugreek		Up to 18.54% when ultrasound assistance is performed	3.23 × 10^5^ g mol^−1^	Galactomannan (73.6%) with 5.5% protein, 0.5% ash, and 10.2% moisture on regular conditions, and 85.89% carbohydrate, 0.85% (db) protein, 5.35% (db) ash, and 7.03% (wb) moisture when extracted with ultrasound aid.	Pseudoplastic behavior with viscosity ≈ 286 mPa·s (30 °C, 170 s^−1^) up to 8.5414 Pa·s (at 1%)	Fenugreek in food formulations is known for its attractive flavor and color, and the gum a uniform, smooth surface that could be of great use in edible films	[56,63,64]
Lamiaceae	*Salvia hispanica*	Chía		10.90%	800–2000 kDa	Anionic heteropolysaccharide, whose defatted seed gum composition was 10.90% lipids, 18.99% fiber, 33.26% protein, 8.28% ash and 8.95% water, while regular chia gum was 26.24% lipids, 28.96% fiber, 25.07% protein, 5.48% ash, and 9.32% moisture	Non-Newtonian behavior with an intrinsic viscosity ∼ 16 dLg^−^^1^	Due to its dietary fiber, flavor-retaining fat contents, and hemicellulose absence, chia gum could play an important role in food processing as a thickener for sensory improvement	[56,65,66]
	*Salvia macrosiphon*	Marmareshk		7.04–12.20%	≈4 × 10^5^ Da	Galactomannan with a 1.78–1.93:1 mannose to galactose ratio, with a composition of 69.01% carbohydrates, 2.08% protein, 11.24% moisture, 9.20% ash, and 30.2% uronic acids		High extraction yield in respect to other gums makes it an appealing new HC, of similar composition, conformation and rheology to conventional guar and xanthan gums, useful in food and pharmaceutical applications	[56,67]
	*Hyptis suaveolens*	Chan–pignut		3.4 ± 0.4%	Total polysaccharides in mucilage = 220 kDa		Non-Newtonian behavior of zero-shear rate viscosity at 0.75% concentration of 1139.79 ± 81.11 Pa·s	Dispersions of 0.25% or greater concentrations show an intermediate behavior between a weak and elastic gel. Interactions of the *H. suaveolens* mucilage with NaCl and sucrose on a pseudoplastic behavior show possible sweet and salty food applications, as a stabilizing, thickening, and gelling agent	[56,68,69]
	*Ocimum basilicum*	Basil		11.46–15.42%		Crude gum had 7.39% moisture, 2.01% protein, 11.55% lipids, 5.89% ash, and 74.19% carbohydrates, while purified gum presented 5.79% moisture, 1.56% protein, 9.71% lipids, 3.32% ash, and 79.62% carbohydrates	Non-Newtonian fluid of 0.230 ± 0.023 Pa·s viscosity under Herschel–Bulkley model	Seeds geographical origin influenced extraction yield of the gum	[56,62,70]
Linaceae	*Linum usitatissimum*	Flaxseed		10.97–12.73%		Commercial flax gums report composition of up to 41% lipids, 28% total dietary fiber, 20% protein, 7.7% moisture, and 3.4% ash	Viscosities of gums extracted at 70 °C and 98 °C were 96.7 vs. 78.8 mPa·s, respectively	Extraction conditions show a higher yield with improved emulsification properties and enhanced emulsion stability when higher temperatures where used, but color is a hindrance of the conditions.	[56,71]
Malvaceae	*Durio zibethinus*	Durian		18% for crude seed gum (light brown), 1.2% for air-dried pure gum and 0.5% for freeze-dried		Pure gum consisted of 17.9% moisture, 29.8% ash, with presence of L-rhamnose, glucose and D-galactose in a 3:9:1 ratio.	Durian gum had a viscosity 65 mPa·s at 1% concentrations, with 29.8°C temperatures at neutrality, but increased slightly with pH from 9 to 10	Although viscosity of the gum was higher at neutral pH, and 1% dispersions have a similar pH to guar gum, durian gums showed a fair stability along a pH range from 2 to 10. A highlight in the gum’s composition is its high zinc content in comparison to other HCs	[56,72]
	*Guazuma ulmifolia*	Mutamba or bay cedar		67.1 g/kg of seeds		*G. ulmifolia* gum consisted of 87.19% total carbohydrates, 10.47% ash, 7.10% fat, 2.93% crude fiber, and 0.89% protein.	Non-Newtonian behavior with a consistency index of 5.11 ± 0.28 Pa·s at 25 °C with concentrations of 1% of G*. ulmifolia* gum	The gum shows good water dispersion and good stability at high temperatures, and is a good carrier and adhesive agent for biological control of pests.	[56,73]
Plantaginaceae	*Plantago ovate*	Isfarzeh or psyllium		4.5–9.2% depending on water temperature for extraction and KOH presence	950–1100 kDa	Psyllium gum and its different fractions predominantly consisted of xylose, (58.2–73.7 mol %), rhamnose (15.1 mol %), arabinose (12.3–24.2 mol %), galactose (3.7–3.8 mol %), and galacturonic acid (0.4–9.7 mol %)	Non-Newtonian behavior with intrinsic viscosity of 3.1–7.4 dL/g in different fractions obtained by higher temperatures or KOH aids.	Secretes three layers of fluid mucilage with similar molecular weight and composition; besides having different possible applications due to water solubility, offers possibilities for biomimetic engineering.	[56,74]
	*Plantago asiatica*	Chinese plantain		13.90%	3.8 × 10^−6^ kDa	*P. asiatica* gum consisted of 82.84% sugar, 0.68% protein, and 20.50% uronic acid and had a monosaccharide composition in a molar ratio of 63.95 Xyl, 15.41 Ara, 2.58 Gal, 1.29 Glc, and 1.00 Rha.	Intrinsic viscosity of the polysaccharide was 5.81 dL/g	Acidic arabinoxylan obtained from *P. asiatica* has a pseudoplastic behavior of weak gelling property that improved with sodium and calcium ions.	[56,75]
	*Plantago major*	Barhang or greater plantain		15.18%	1.2 × 10^6^ Da.	*P. major* gum was composed of 82.85% carbohydrates, 6.80% ash, 6.66% protein, 3.69% moisture, and trace amounts of fat.	Intrinsic viscosity in deionized water at 25 °C was 14.24 ± 0.61 dL/g	In addition to being a high-yield, economically interesting gum for stabilizing foods, *P. major* showed appealing bioactive characteristics in terms of total phenol contents, total flavonoid content, and antioxidant activity in amounts of 76.79 mg GAE/g dry, 97.8 mg g^−1^ and 915.54 g/mL, respectively	[56,76]
Rosaceae	*Cydonia oblonga*	Quince		16.29%		*C. oblonga* gum consisted of 71.6% carbohydrates, 12.59% ash, 3.16% fat, 2.81% protein, and 9.84% moisture.	Apparent viscosity of the gum was approximately 52.4 mPa·s	Ultrasound-assisted extraction significantly increases purity, extraction yield, and viscosity of *C. oblonga* gums	[56,77]
	*Prunus cerarus*	Dwarf or sour cherry	Exudate		0.56 × 10^5^ g/mol	Arabinogalactan including arabinose, xylose, galactose, glucopyranosyluronic acid, rhamnose, and mannose.	Intrinsic viscosity of *P. cerasus* and related *P. avium* were 3.84 and 7.08 dl/g, respectively	Harvest season and species strongly affect molar mass distribution and polydispersity of Prunus gums, but similar magnitudes of molecular weight to arabic, karaya, and tragacanth exudate gums suggests a possible industrial application.	[56,78]
Annonaceae	*Annona crassiflora*	Araticum	peel						[56]
Irvingiaceae	*Irvingia gabonensis*	African mango	seeds	79% mass recovery	1.5 × 10^6^ g/mol	Polysaccharide prevailing fraction made by 61.72% galactose, 18.8% arabinose, 8.7% rhamnose, 9.1% galacturonic acid, 1.1% glucose, and 0.5% glucuronic acid.	Intrinsic viscosity at infinite ionic strength was 4.9 dL/g	The arabinogalactan contains a small portion of neutral sugars and uronic acids that give rise to polyelectric properties that confer stabilizing properties to the gum.	[79,80]
Lauraceae	*Beilschmiedia obscura*	Khan		6.60%			Apparent viscosity ≈ 0.70 ± 0.06 dL/g		[79,81]
Malvaceae	*Triumfetta cordifolia*	Nkui	Bark				Apparent viscosity ≈ 18.29 ± 0.64 dL/g		[79]
	*Corchorus olitorius*	Lalo–nkeling nkeling	leaves				Apparent viscosity ≈ 2.11 ± 0.39 dL/g		
	*Adansonia digitata*	Baobab–bocco					Apparent viscosity ≈ 1.64 ± 0.06 dL/g		
Pedaliaceae	*Ceratotheca sesamoides*	Gougdo or false sesame					Apparent viscosity ≈ 3.04 ± 0.39 dL/g		
Phyllantaceae	*Bridelia thermifolia*	Kelly	bark				Apparent viscosity ≈ 2.47 ± 0.17 dL/g		
Cucurbitaceae	*Cucurbita moschata*	Butternut squash	peel	10%	26–96 kDa prevailing higher molecular weight of *C. moschata* pectin	Predominant composition in neutral sugars as: glucose, rhamnose, galactose, arabinose, and mannose	Gum’s intrinsic viscosity from 0.085 to 0.027 L/g when temperature rises, and pectin around 4.5 dL/g		[82,83]
Rosaceae	*Prunus* spp. (*P. scoparia, P. dulcis*, *P. armeniaca, P. spinosa*, *P. caroliniana*, *P. laurocerasus*, *P. virginiana*, *P. persica*)	Persian gum (from wild almond), cherries, plums, peaches.	Exudates						[84]
Pinaceae	*Larix gmelinii*	Gmelin Larch wood	exudates					*P. alba* gum was useful as a polyelectrolyte stabilizing alginate–calcium–chitosan beads, as a fish oil excipient.	[30,85]
Fabaceae	*Prosopis alba*	White carob	exudate						[86]
Solanaceae	*Solanum betaceum*	Tamarillo	puree						[87]
Solanaceae	*Solanum nigrum*	Black nightshade	fruit						[88]
	*Solanum surattense*	Yellow berried nightshade	fruit						
	*Solanum indicum*	Poison berry	fruit						

^1^ Most common names are listed for practical purposes, but these may vary depending on the location.

**Table 2:** Nonconventional starch sources, botanical origin, and thermal properties.

Geographical Origin	Taxonomy			Plant Structure	Amylose Content (%)	Granule Characteristics		Thermal Properties				References
	**Family**	**Spp.**	**Common Name**			Morphology	Size (μm)	ΔH (J/g)	To (°C)	Tp (°C)	Tc (°C)	
Fresh and saline environments	Chlorellaceae (Chlorophyta Phyllum)	*Chlorella sorokiniana*	Microalgae	Whole biomass	17	Ovals and spheres	1.5	5.49	24	69	97	[108]
South America (Andean Region)	Fabaceae	*Erythrina edulis*	Chachafruto, basul	Seed	14.14	Oval, spherical, truncated	40	18.54	65.26	70.48	75.79	[114]
Central Andean Region	Amaranthaceae	*Chenopodium quinoa*	Quinoa	Seed	15.7	Semi–spherical cumulus	Submicrons		49.69	60.41	71.14	[115]
Mediterranean	Fabaceae	*Lathyrus sativus*	Grass pea (cv. Derek, Krab)	Seed	35.2–35.8	Oval–ellipsoid	6–40	12.6	60.2–61.3	67.5–68.5	74.2–74.6	[111]
Mexico	Nyctaginaceae	*Okenia hypogaea*	Okenia	Seed	26.1	Round–oval	1–3	11.94		71.3		[110]
Gondwana/South hemisphere	Araucariaceae (Conifer)	*Araucaria brasiliensis*	Paraná, Brazil pine	Seed	22.4–25	Round–oval with a central hilum	10–25	8	63.6	63.4–68.5	75.8	[116,117]
Gondwana/South hemisphere	Araucariaceae (Conifer)	*Araucaria araucana*	Pewen, Chilean pine	Seed	17.3	Round–oval with a central hilum	8.4		61	66.6	73.5	[117]
Central Andean Region	Oxalidaceae	*Oxalis tuberosa*	Oca, ibia	Stem tuber	27.6	Primarily cylindrical, ellipsoid, and oval	6.99–38.2	9.66	50.26	55.17	63.91	[118,119]
Central Andean Region	Bassellaceae	*Ullucus tuberosus*	Olluco, chugua, ruba	Tuber	26.49	Irregular, primarily ellipsoid, oval, conical and prismatic	4.48–32.64	10.23	52.81	58.93	67.88	
Central Andean Region	Tropaeolaceae	*Tropaeolum tuberosum*	Isaño, mashua, cubio	Tuber	27.44	Oval, spherical, and truncated	4.45–22.9	9.78	51.85	56.92	65.22	
Central Andean Region	Apiaceae	*Arracacia xanthorrhiza*	Arracacha	Taproot	35.7–39	Round, smaller polygonal or truncated	5.36–23.80	6.1–8.8	53–54.9	57.8–59.1	70.4–73.9	[118,120]
Mexico	Araceae	*Xanthosoma yucatanensis*	Makal, malanga	Rhizome	23.6	Round	12.4	14.9	72.6	78.5	84.2	[121]
Central America	Araceae	*Xanthosoma sagittifolium*	Arrowleaf elephant ear, taro, bore	Corm	16–24	Round	2.8–50	4–15	66–83	69–85	79–90	[122]
South America	Brassicaceae	*Lepidium meyenii*	Maca	Taproot	20.5	Oval and irregular	5.8–14.9	6.22	45.7	47.7	51.16	[123]
South and Central America	Cannaceae	*Canna edulis*	Achira, edible canna	Tuber	21.24–31.71	Disk–oval	24.4–102.53	10.62–13.55	61.16–63.06	63.34–65.64	67.87–71.09	[124]
Central Asia	Poaceae	*Panicum miliaceum*	Proso (broomcorn) millet	Seed	0.75–30.7	Round (smaller), polygonal (larger)	1.66–11.66	15.6–28.1	68.4–75.6	72.1–79.6	68–83.5	[125]
Southern Asia	Musaceae	*Musa acuminata*	Gros Michel banana	Fruit		Ellipsoidal	16.6–48.53	44.62	*33.59*	48.36	64.37	[126]
Melanesia	Araceae	*Cyrtosperma merkusii*	Giant swamp taro	Corm	24.97	Round and spherical	12.5					[127]
Eastern China and Japan	Fabaceae	*Apios fortunei*	Hodo—potato bean	Root tuber		Spherical, polygonal, ellipsoidal	5–30		62.1	68.3–75.4	83.9	[128]
Andean Region	Fabaceae	*Pachirrhizus ahipa*	Andean yam bean	Tuberous root	13.71	Round and polygonal	7.95	9.1	65.19	69.13		[129]
Central America	Sapotaceae	*Pouteria campechiana*	Cupcake fruit, canistel	Fruit and seed	16.63–33.65	Oval to bell–shaped	4.92–30.15	8.43–11.06	59.75–67.30	65.97–73.34	77.79–82.92	[130]
Eastern Asia	Polipodyaceae (Fern)	*Drynaria roosii*	Gu-sui-bu	Rhizome	30.01	Elliptical, spherical, irregular	15.15					[131]
Eastern Asia	Theaceae	*Camellia sinensis*	Tea	Seed	27.06–33.17	Flat spherical or oval	9	12.8–12.94	60.84–68.56	64.99–76.03	70.47–82.74	[132]
Indo-China	Fabaceae	*Vigna umbellata*	Rice bean	Seed	27.29	Round (smaller), Oval, and elliptical (larger)	659.8 nm	N/A				[133]
Southern Japan and Korea	Fagaceae	*Castanopsis cuspidate*	Japanese chinquapin	Fruit	56.1	Ellipsoid to polygonal or angulate	8.13–20	14.1	56	61.3	72.4	[134]
Mexican cross from Philippines cultivar	Anacardiaceae	*Mangifera indica cv. Ataulfo*	Mango	Stenospermocarpic unripe fruit			10–15					[112]
Tropical Americas	Bixaceae	*Bixa orellana*	Annato	Byproduct seed	27.8	Oval, flake-like	17.2		64.7			[135]

Predominant granule size and thermal properties’ ranges are shown as a representation of each species (Spp.). Thermal properties depicted include To: gelatinization onset temperature, Tp: gelatinization temperature peak, Tc: gelatinization conclusion temperature, and ΔH, gelatinization enthalpy. Synonyms for some species include *A. brasiliensis = Araucaria angustifolia, P. campechiana = Lucuma nervosa*.
